# Targeted Antimicrobial Agents as Potential Tools for Modulating the Gut Microbiome

**DOI:** 10.3389/fmicb.2022.879207

**Published:** 2022-07-07

**Authors:** Shuli Chou, Shiqing Zhang, Huating Guo, Yung-fu Chang, Wenjing Zhao, Xiangyu Mou

**Affiliations:** ^1^Center for Infection and Immunity Studies, School of Medicine, Shenzhen Campus of Sun Yat-sen University, Shenzhen, China; ^2^Department of Population Medicine and Diagnostic Sciences, College of Veterinary Medicine, Cornell University, Ithaca, NY, United States; ^3^Guangdong Provincial Key Laboratory of Colorectal and Pelvic Floor Diseases, The Sixth Affiliated Hospital, Sun Yat-sen University, Guangzhou, China

**Keywords:** berberine, polyphenols, bacteriocins, antimicrobial peptides, phage therapy, targeted drug delivery system, pathobionts, microbiome editing

## Abstract

The gut microbiome plays a pivotal role in maintaining the health of the hosts; however, there is accumulating evidence that certain bacteria in the host, termed pathobionts, play roles in the progression of diseases. Although antibiotics can be used to eradicate unwanted bacteria, the side effects of antibiotic treatment lead to a great need for more targeted antimicrobial agents as tools to modulate the microbiome more precisely. Herein, we reviewed narrow-spectrum antibiotics naturally made by plants and microorganisms, followed by more targeted antibiotic agents including synthetic peptides, phage, and targeted drug delivery systems, from the perspective of using them as potential tools for modulating the gut microbiome for favorable effects on the health of the host. Given the emerging discoveries on pathobionts and the increasing knowledge on targeted antimicrobial agents reviewed in this article, we anticipate targeted antimicrobial agents will emerge as a new generation of a drug to treat microbiome-involved diseases.

## Introduction

The mammalian gut microbiome plays a pivotal role in maintaining stable gut physiology and organism homeostasis. The roles of individual bacterial species in the human gut microbiome have been studied since early 1900, and have been greatly accelerated by the advance in sequencing technology since the mid-2000s. There is accumulating evidence that some commensal species play beneficial roles in maintaining health in the host while some other species, termed pathobionts, play in detrimental ways.

**Figure 1:**
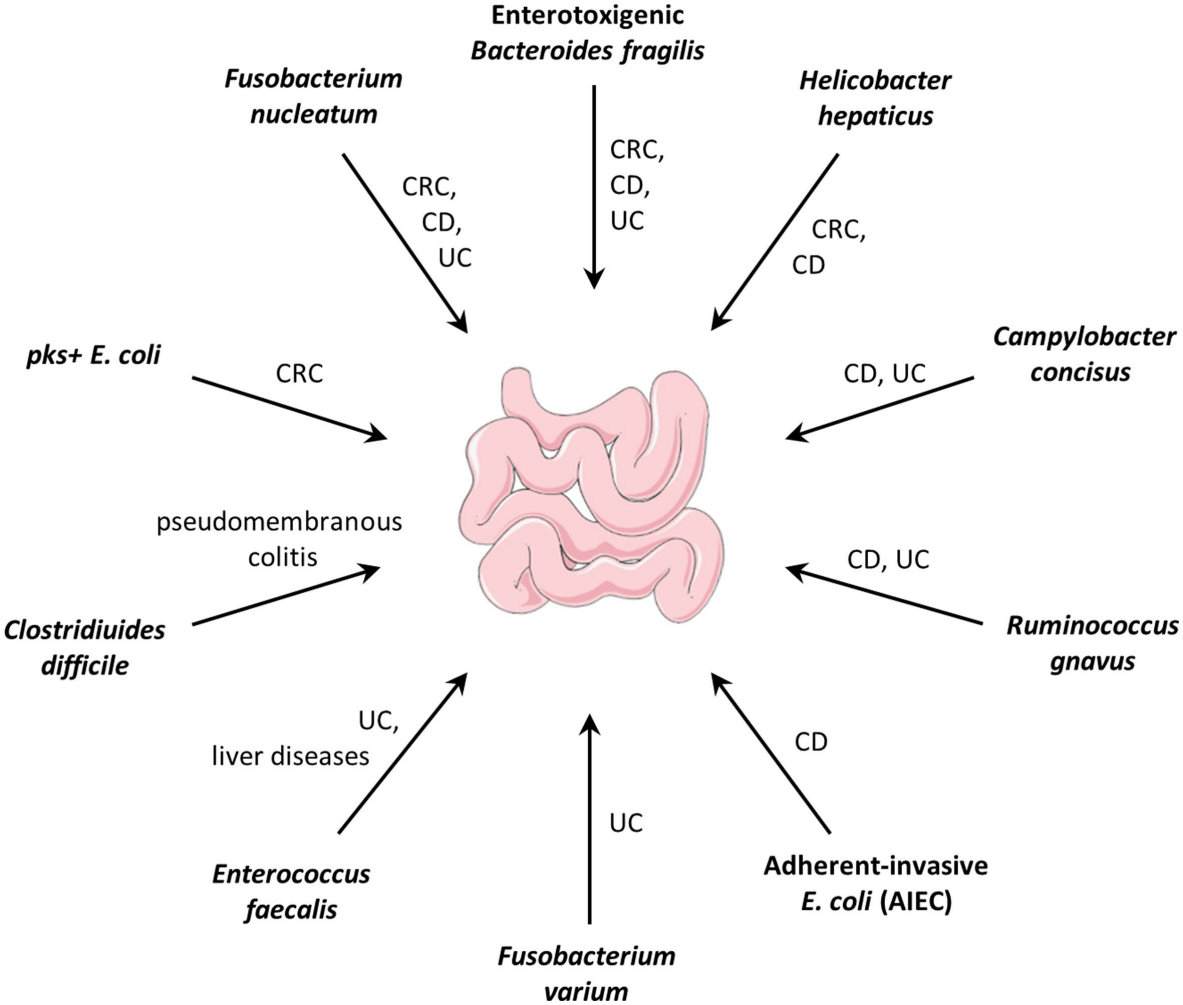
Gut pathobionts and their related diseases. CRC: colorectal cancer; CD: Crohn’s disease; UC: ulcerative colitis.

Pathobionts often refer to symbiotic bacteria with pathogenic potential that contribute to the progression of a disease, but have not yet been recognized as pathogens (see review: Chow et al., 2011; Gill and Rosenbaum, 2020; Chandra et al., 2021). For example, *Fusobacterium nucleatum*, an oral symbiotic bacterium was demonstrated as a pro-carcinogenic bacterium in colorectal cancer (CRC; Brennan and Garrett, 2019; Yachida et al., 2019; Slade, 2021). Enterotoxigenic *Bacteroides Fragilis* (ETBF) which is associated with inflammatory bowel disease and CRC in humans, contributes to colitis and carcinogenesis in mouse models (Chung et al., 2018; Cao et al., 2021). The *pks^+^ Escherichia coli* that produces the genotoxic colibactin was shown to drive tumorigenesis in mouse models, human mini-guts, and CRC patients (Pleguezuelos-Manzano et al., 2020; Iftekhar et al., 2021). Gut pathobionts and their related diseases are summarized in Figure 1.

For pathobiont-promoted diseases, pathobionts can serve as therapeutic targets. Antibiotics are the first-line consideration for treating bacterial infection; however, antibiotic treatment has many side effects, including secondary infections, digestive problems, and the emergence of antibiotic-resistant bacteria. Therefore, there is a great need for tools that can be used to modulate the microbiome more precisely. Such tools would make pathobionts “manageable” and their related diseases preventable with minimal side effects. In this review, we summarized five categories of targeted antimicrobial agents that they are promising to be developed as tools for modulating the gut microbiome to achieve favorable outcomes in the host. We first reviewed narrow-spectrum antimicrobial agents made by plants and microorganisms, followed by more targeted antimicrobial agents including synthetic antimicrobial peptides, phage, and targeted drug delivery systems.

## Narrow-Spectrum Antimicrobial Agents From Plants

Narrow-spectrum antimicrobial agents are active on a limited range of bacteria while leaving a wide range of bacteria unharmed. For example, berberine, a quaternary ammonium alkaloid made by plants including Chinese goldthread (*Coptis chinensis* Franch.), showed antimicrobial activity on *Staphylococcus aureus, Streptococcus pneumoniae, Enterococcus faecium, Bacillus dysenteriae, Shigella flexneri, and Helicobacter pylori*, and showed no antimicrobial activity on a wide range of bacteria that span across multiple phyla, including *Staphylococcus epidermidis, Escherichia coli, Klebsiella pneumoniae, Acinetobacter Baumanni, Enterobacter cloacae, Proteus mirabilis, Bifidobacterium longum and Lactobacillus casei* (Lin Yuan and Jian-Dong, 2018).

Not surprisingly, the oral intake of berberine resulted in an altered microbiome in the hosts. Interestingly, in addition to the alteration of the microbiome, berberine and its derivatives were shown to have broad effects on the health or progression of diseases in the hosts, suggesting the link between the effects of berberine and the altered microbiome.

### Berberine as a Tool for Modulating the Gut Microbiome

Although berberine sometimes affects the host’s health by directly interacting with host targets (Ren et al., 2021), there is emerging evidence that berberine’s effects are mediated by the gut microbiome (see review: Zhang et al., 2020a; Cheng et al., 2021). For example, berberine attenuated choline-induced atherosclerosis in a mouse model by down-regulating the bacterial production of trimethylamine, a pro-atherosclerosis molecule produced by the gut microbiome (Li et al., 2021). In another mouse model, berberine ameliorated the ovariectomy-induced anxiety-like behaviors by enriching the quote-generating gut microbiome (Fang et al., 2021a). Further, a study on human revealed the antidiabetic effect of berberine on type 2 diabetes is mediated by the inhibition of *Ruminococcus bromii*, which break down a type of secondary bile acid that contributes to glycemic control (Zhang et al., 2020b).

### Other Plant Ingredients

Besides berberine, polyphenols, a type of compound made by plants, also showed narrow-spectrum antimicrobial activity *in vitro* (see review: Gonzalez-Lamothe et al., 2009). Similar to berberine, oral intake of polyphenols also has beneficial effects on multiple diseases of the host while altering the gut microbiome in the host. These diseases include colitis-associated colorectal cancer, cardiovascular disease, and obesity (Gowd et al., 2019; Martinet et al., 2019; Jennings et al., 2021; Rufino et al., 2021; Zhao and Jiang, 2021). Given the accumulating association between polyphenols’ effects on health and the gut microbiome modulation, it is likely that the beneficial effects of polyphenols are mediated by the gut microbiome, although further study is required to reveal the roles of the gut microbiome in these effects.

In addition to determining ingredients like berberine and polyphenols, some plant extracts showed narrow-spectrum antibacterial activity *in vitro.* For example, ethanolic extracts of *Passiflora mollissima*, which are rich in several phytochemicals, including alkaloids, saponins, essential oils, carotenoids, and anxiolytic, showed selective activity against *in vitro* cultured strains of *Streptococcus mutans, Streptococcus oralis,* and *Streptococcus sanguiniss* (Adrián Calderon et al., 2019), although the active ingredients are yet to known.

## Narrow-Spectrum Antimicrobial Agents From Microorganisms

Antimicrobial peptides (AMPs) are produced by a broad spectrum of organisms including microorganisms, plants, insects, and vertebrates. Animal AMPs are essential components of the innate immune system, and often have broad-spectrum antimicrobial activity (Ostaff et al., 2013; Sivieri et al., 2017; Aresti Sanz and El Aidy, 2019). In contrast, bacterial AMPs, termed bacteriocins, often exhibit a limited spectrum of antimicrobial activity, effective on bacteria that are phylogenetically related to the bacteriocin-producing bacteria (Meade et al., 2020).

Most bacteriocins kill target bacteria by pore formation on the membrane of the victims (Kumariya et al., 2019), and other mechanisms include killing by condensation of genomic DNA or inhibition of cell wall synthesis (Mengxin Geng and Smitha, 2018; Qin et al., 2019). Bacteriocins are mainly categorized into three classes, based on their structural and physicochemical properties: Class I, Class II, and Class III. Class I bacteriocins belong to ribosomally synthesized and post-translationally modified peptides (RiPPs). They are also known as lantibiotics because they contain the unusual amino acids, lanthionine, and methyllanthionine. Class I bacteriocins often exhibit broad-spectrum antimicrobial activities. In contrast, Class II bacteriocins are predominantly unmodified peptides (Soltani et al., 2021), and often showed narrow-spectrum antimicrobial activity (Moll et al., 1999; Ríos Colombo et al., 2019). They are first synthesized as prebacteriocins with an N-terminal leader, and the leader is later removed during the process of secretion. For example, rhamnocin 519 showed a narrow-spectrum antibacterial activity against *Listeria monocytogenes* and *S. aureus* (Jeong and Moon, 2015). Class III bacteriocins are heat-labile, high molecular weight antibacterial proteins. For example, geobacillin 26 has a narrow antibacterial spectrum against some thermophilic bacteria (Vaičikauskaitė et al., 2019). To target pathobionts in the gut with minimum collateral damage to gut normal flora, narrow-spectrum bacteriocins are more favorable than broad-spectrum bacteriocins. A list of Class II bacteriocins that exhibit the narrow spectrum of antimicrobial activities is summarized in Table 1.

**Table 1:** Selective antimicrobial activity of class II bacteriocins.

Bacteriocins	Sensitive bacteria	Resistant microorganism	Origin	References
Piscicolin	*Listeria monocytogenes*	Gram-negative bacteria	*Carnobacterium* *maltaromaticum*	Martin-Visscher et al., 2011
Mesenterocin	*Listeria monocytogenes* *Carnobacterium divergen* *Enterococcus faecium* *Lactobacillus plantarum* *Lactobacillus sakei* *Pediococcus acidilactici* *Pediococcus pentosaceus*	*Lactococci*, *Lactobacilli*	*Leuconostoc* *mesenteroides*	Osmanagaoglu and Kiran., 2011
Leucocin	*Listeria monocytogenes* *Lactobacillus sakei* *Lactobacillus formosensis* *Lactococcus lactis* *Entercoccus durans*	*Weissella hellenica* *Lactococcus garvieae* *Staphylococcus aureus* *Acinetobacter baumannii* *Escherichia coli* *Bacillus thuringiensis* *Bacillus subtili*	*Leuconostoc* *pseudomesenteroides*	Chen et al., 2018
Curvacin	*Listeria monocytogenes* *Lactobacillus paracasei* *Lactobacillus sakei* *Enterococcus faecium* *Bacillus cereus*	*Staphylococcus aure* *Salmonella typhimurium* *Klebsiella pneumonia* *Escherichia coli* *Saccharomyces cerevisiae* *Candida pseudotropicalis* *Penicillium roqueforti*	*Lactobacillus curvatus* *Lactobacillus sakei*	Ahmadova et al., 2013
Curvaticin	*Staphylococcus aureus* *Enterococcus faecalis* *Listeria monocytogenes*	*Lactococcus lactis*	*Lactobacillus curvatus*	Bouttefroy et al., 2000
Garvicin ML	*Lactobacillus casei,* *Lactobacillus sakei,* *Pediococcus acidilactici, Enterococcus faecium*	*Pseudomonas fluorescens,* *Escherichia coli,* *Salmonella paratyphi*	*Lactococcus garvieae*	Borrero et al., 2011
Leucocyclicin Q	*Lactococcus lactis subsp. lactis,* *Lactobacillus sakei subsp. sakei, Weissella paramesenteroides,* *Pediococcus dextrinicus*	*Staphylococcus aureus subsp. aureus*	*Leuconostoc mesenteroides*	Masuda et al., 2011
Lactocyclicin Q	*Pediococcus extrinicus,* *Lactococcus lactis subsp. lactis, Lactobacillus sakei subsp. sakei,* *Bacillus coagulans*	*Streptococcus mutans*	*Lactococcus sp. strain*	Masuda et al., 2011
Carnocyclin A	*Brochothrix campestris,* *Enterococcus faecalis,* *Lactococcus lactis subsp. lactis*	*Escherichia coli,* *Pseudomonas aeruginosa,* *Salmonella enterica*	*Carnobacterium maltaromaticum*	Borrero et al., 2011
Avicin A	*Carnobacterium divergens, Enterococcus avium,* *Enterococcus faecalis,* *Leuconostoc lactis*	*Lactobacillus plantarum,* *Leuconostoc gelidum,* *Pediococcus acidilactici*	*Enterococcus avium*	Birri et al., 2010
Laterosporulin10	*Bacillus subtilis,* *Staphylococcus aureus, Mycobacterium tuberculosis*	*Bacillus subtilis,* *Vibrio cholerae,* *Escherichia coli,* *Pseudomonas aeruginosa*	*Brevibacillus sp. strain*	Baindara et al., 2016

In addition to bacteriocins, many fungi and bacteria secrete antimicrobial secondary metabolites. Unlike many broad-spectrum antibiotics that are used in preventing infectious diseases, some secondary metabolites exhibit selective antimicrobial activity, some of which are summarized in Table 2.

**Table 2:** Selective antimicrobial activity of bacterial secondary metabolites.

Metabolites	Sensitive bacteria	Resistant microorganism	Origin	References
Tyromycin A	*Bacillus subtilis*	MRSA *Staphylococcus aureus, Micrococcus luteus*	*Skeletocutis sp.*	Chepkirui et al., 2019
Aspergyllone	*Candida* *parapsilosis*	*Pseudomonas aeruginosa* *Staphylococcus aureus* *Escherichia coli* *Candida albicans,* *Candida krusei* *Candida glabrata,* *Candida utilis*	*Aspergillusniger* *Tiegh*	Padhi et al., 2020
Anthraquinone dimmers (compounds 1 and 2)	*Staphylococcus* *aureus*	*Escherichia coli* *Salmonella typhimurium* *Klebsiella aerogenes* *Enterobacter cloacae* *Pseudomonas aeruginosa*	unidentified fungal strain INF16–17	Li et al., 2019
*P. herquei* extract	*Staphylococcus aureus* MRSA	*Candida albicans* *Candida glabrata* *Candida krusei* *Candida neformans* *Aspergillus fumigates* *Escherichia coli* *Pseudomonas aeruginosa* *Mycobacterium intracellulare*	*Penicillium herquei*	Ferreira et al., 2017
Glycerol 1-hydroxy-2,5-dimethyl benzoate 1	MRSA	*Mycobacterium tuberculosis* *Bacillu subtilis* *Pseudomonas aeruginosa* *Candida albicans*	*Verrucosis pora sp. strain MS* 100047	Huang et al., 2016
*Candida cibarius* methanol extract	*Enterococcus* *faecalis*	*Staphylococcus aureus* *Shigella sonnei* *Salmonella enteritidi* *Yersinia enterocolitica* *Bacillus cereus* *Listeria monocytogenes*	*Cantharellus cibarius Fr.*	Kozarski et al., 2015

In contrast to berberine and polyphenols, the effects of oral intake of bacteria-derived antimicrobial agents on the alteration of the host-microbiome are not extensively studied; however, it would be interesting to test these narrow-spectrum antimicrobial agents from microorganisms as potential tools for modulating gut microbiome to achieve beneficial effects on the health of the host.

## Synthetic Antimicrobial Peptides

Inspired by bacteriocins, synthetic AMPs have emerged since 1992 containing only leucine and lysine residues. The study field of synthetic AMPs was first focused on the observation of the correlation between the structural properties and the antimicrobial activities of AMPs (Fang et al., 2021b). During the past decade, three main parameters of AMPs have been revealed to play roles in their antimicrobial activities, which are (1) hydrophobicity, (2) cationic number, and (3) secondary structure. (1) Hydrophobicity contributes to the membrane-binding driving force, which is the main cause of the damage to target cell membranes. Hydrophobicity often determines antimicrobial potency and cell selectivity. For example, leucine and the more hydrophobic isoleucine are isomers. The interchange of them does not alter the structure of the peptide but increases the hydrophobicity. Accompanied by the increase of hydrophobicity, the antibacterial spectrum changed from Gram-negative only to both Gram-negative and Gram-positive bacteria, demonstrating hydrophobicity is one of the key parameters in determining the selectivity of bacteria (Chou et al., 2016). (2) Cationic number of AMPs is determined by cationic residues like arginine, lysine, and histidine, and the cationic number determines the affinity of AMPs to the negatively charged lipid head groups on the outer membrane of bacteria. (3) Secondary structures of AMPs are often determined by key amino acids including cystine and glycine. Cysteine residues, which can form disulfide bonds, are the prerequisite for cyclization and β-sheet (de Leeuw et al., 2007). Glycine, the smallest hydrophilic amino acid, determines the flexibility of local conformation of AMPs, which contributes to enhanced activity against Gram-negative bacteria (Wang et al., 2015). Notably, the selectivity of AMPs is often determined by the three main parameters mentioned above as well as bacterial factors, which provides a rationale for designing more targeted AMPs, termed specifically targeted antimicrobial peptides (STAMPs; Eckert et al., 2006; Yount et al., 2019).

**Figure 2:**
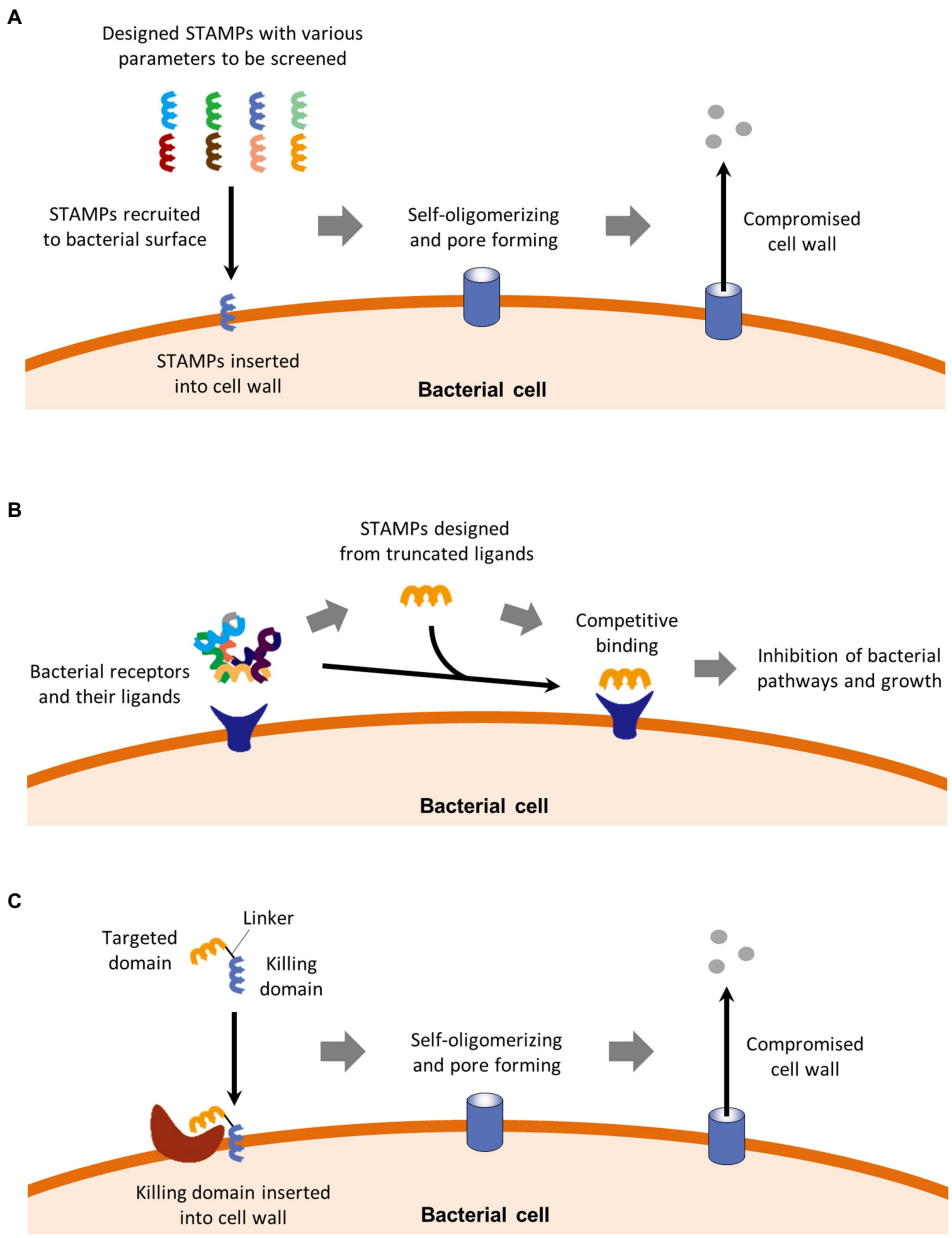
STAMPs can be divided into three categories. **(A)** Canonical STAMPs are usually discovered by screening a batch of peptides with various parameters including hydrophobicity, cationic number, and secondary structure. These parameters, as well as some bacterial factors, determine the affinity between the peptides and bacterial surfaces. Canonical STAMPs kill bacteria by insertion into the cell membrane, followed by self-oligomerization, pore formation, and cell membrane perturbation. **(B)** Peptide ligands as STAMPs. This category of STAMPs kills specific bacteria by competitively binding bacterial receptors of physiological importance against the natural ligands of the receptors, resulting in inhibition of important bacterial pathways and subsequential inhibition of bacterial growth. **(C)** Composite STAMPs consist of a targeted domain and a killing domain. The targeted domain binds to specific motifs on targeted bacteria, which directs the killing domain to kill bacteria in the same way as canonical STAMPs.

For the past decade, STAMPs have been studied to kill specific pathogens while not affecting the normal flora (Phulen Sarma et al., 2018). As a peptide, STAMPs are highly flexible for adopting structural and functional amino acid groups (Roncevic et al., 2019), so that they can achieve a much narrower spectrum than natural narrow-spectrum antimicrobial agents reviewed above. STAMPs can be divided into three categories which are, respectively, reviewed below and illustrated in Figure 2, based on different rational designing strategies.

### Canonical STAMPs

Canonical STAMPs kill bacteria by insertion into the cell wall surface, followed by self-oligomerization pore-forming, and perturbations in the cell wall (Guo et al., 2015; Maraming et al., 2019). The selectivity or antibiotic spectrum of STAMPs is mainly determined by the three main parameters. To better assist the designing of STAMPs with the desired antimicrobial spectrum, a comprehensive AMP database has been developed (Wang et al., 2009). Based on the AMP database, many STAMPs have been developed, including STAMPs against *E. coli*, *Salmonella pullorum*, *Pseudomonas aeruginosa,* and *S. aureus* (Mishra and Wang, 2012; Chou et al., 2019, 2021; Roncevic et al., 2019). In a mouse model, a pH-dependent STAMP was demonstrated to kill *H. pylori* in an acidic environment in the stomach with minimal toxicity to commensal bacteria in the gut (Xiong et al., 2017).

### Peptide Ligands as STAMPs

Besides the designing strategy based on the AMP database, an alternative designing strategy has been developed. In this strategy, STAMPs were obtained by truncating the sequences from the natural protein ligands of the targeted bacterial receptor of important physiological importance. The resulting STAMP then competitively inhibit the receptor and its downstream pathways. For example, the sequence of a STAMPs that binds to *F. nucleatum* FadA, was shortened from mammalian E-cadherin, the natural ligands of FadA (Rubinstein et al., 2013). The E-cadherin mimicking STAMP successfully inhibited the FadA-dependent *F. nucleatum*-induced tumor and inflammation in mouse models.

### Composite STAMPs

The third designing strategy of STAMPs involves conjugating a preselected targeted peptide with a wide-spectrum AMP domain together (Lei et al., 2021), resulting in composite STAMPs with a targeted domain and a killing domain. For example, bacterial pheromones have been used as targeted domains in STAMPs against *S. mutans*, *S. aureus*, *Enterococcus faecalis,* and/or *Streptococcus agalactiae* (Qiu et al., 2003; Huo et al., 2018; Li et al., 2020; Xu et al., 2020). Furthermore, a cell wall precursor lipid II binding peptide, screened from a library of phage, was used as a targeted domain in a STAMP that specifically killed some clinic-isolated strains of vancomycin-resistant bacteria (Hart et al., 2017).

### Strategies to Improve the Stability of STAMPs in the Gut

Although in two clinical trials, AMPs have been used to eliminate specific microorganisms in oral cavity and stomach with minimal impact on other commensal bacteria (Sullivan et al., 2011; Xiong et al., 2017), there are no report regarding AMPs targeted on specific bacteria in intestine and colon. One of the challenges in utilization of STAMPs is the proteolytic degradation of STAMPs that occurs in the digestive systems (Hashemi et al., 2018; Bhattacharjya and Straus, 2020). Several strategies have been exploited to improve the stability of AMPs in the gut. Coating is a frequently used strategy. For example, an AMP aiming to treat *Clostridioides difficile* infection is coated with a layer of pectin and hydroxypropyl methyl cellulose, which can protect the AMP against proteolytic enzymes from intestinal digestive enzymes, and release the AMP when encountering pectinolytic enzymes in the colon (Ugurlu et al., 2007). Another strategy is to design STAMPs that are capable to self-assemble into nanoscale particles, which exhibit high stability against enzymatic degradation (Eskandari et al., 2017; Chen and Zou, 2019; Tan et al., 2021). Besides, the introduction of D-amino acids, cyclization, amidation, or acetylation of the terminal regions are also applied to improve proteolytic stability (Dijksteel et al., 2021).

## Utilization of Bacteriophage

### Utilization of Naturally Occurring Bacteriophages

Bacteriophages attach to the very specific receptors on the surface of bacteria such as lipopolysaccharide, lipoteichoic acid, capsular polysaccharide, flagella, and pili before killing them (Hsu et al., 2021), and therefore is a highly targeted antimicrobial agent (Carasso et al., 2020). Phages have been historically used to eradicate bacterial pathogens and were recently used as a potential strategy to treat diseases by the precise killing of target bacteria (Mccarville et al., 2016; Wahida et al., 2021; Zhang et al., 2021). For example, Duan *et al* reported the bacteriophage that targets cytolytic *E. faecalis* decreased cytolysin in the liver and abolished ethanol-induced liver disease in humanized mice (Duan et al., 2019; Colakoglu et al., 2020).

Phage is so selective on targets that a phage strain is usually effective on only one strain of a bacterial species; however, the mixture of multiple phage strains, termed phage cocktails, provide a solution to achieve desired-spectrum phage therapies (Villarroel et al., 2017; Gordillo et al., 2019). For example, an optimized 4-phage cocktail against *Clostridium difficile* eradicated *C. difficile* in 24 h, without affecting commensal gut microbes (Nale et al., 2018).

### Phage-Guided Therapies

Besides the utilization of phages alone, phages have been used as a motif to synthesize phage-guided antimicrobial agents (Gu Liu et al., 2020; Morrisette et al., 2020). For example, the antibiotics linezolid conjugated with a lytic phage, was more effective than each part alone in treating MRSA infections of diabetic foot ulcers in an *S. aureus* infection murine model (Chhibber et al., 2013). Phage was also used in guiding silver nanoparticles in targeting *F. nucleatum* (Xue Dong et al., 2020).

## Targeted Drug Delivery System

The concept of a targeted drug delivery system is frequently used in anti-cancer drugs. A targeted drug delivery system usually consists of a targeted unit and a cargo unit. The targeted units offer high ligand-binding efficiency to the targeted tissue or cells and the cargo units are the bioactive drugs, sometimes loaded inside vehicles like nanoparticles and liposomes (Hussein and Abdullah, 2021). Recently, a targeted drug delivery system has been also employed in the field of specific antimicrobial agents. In a recent study, an intracellular antibiotic delivery system has been developed. The delivery system is composed of three parts: (1) mesoporous silica nanoparticles loaded with gentamicin, (2) lipid bi-layer envelops that would disseminate upon contact of *S. aureus* hemolysins, and (3) *S. aureus*-targeting domain truncated from a previously reported AMP, ubiquicidin 29–41. This delivery system works well in killing *S. aureus* and eliminating the *S. aureus*-induced inflammation in a mouse model (Yang et al., 2018). In another drug delivery system, lipid nanoparticles loaded with antibiotic was conjugated with an *S. aureus*-targeting antibody. The resulting system showed enhanced *in vitro* bactericidal activity against *S. aureus* both in planktonic and biofilm forms (Le et al., 2021).

## Discussion

The knowledge of narrow-spectrum antimicrobial agents has been greatly advanced in the past decade (see review: Fong et al., 2016; Maxson and Mitchell, 2016; Romani-Perez et al., 2017; George Kerry et al., 2018; Riglar and Silver, 2018; Alm and Lahiri, 2020; Altarac et al., 2021; Avis et al., 2021; Fuenzalida et al., 2021). Notably, some narrow-spectrum antimicrobial agents have entered clinical trials, mainly to combat bacteria associated with antibiotic resistance, such as *Acinetobacter baumannii, S. aureus, P. aeruginosa, and E. coli* (see review: Alm and Lahiri, 2020). In this article, we reviewed narrow-spectrum antimicrobial agents from the aspect of using them as a microbiome-modulating tool especially to limit pathobionts in the gut.

Among all natural narrow-spectrum antibiotic agents, berberine is the most well studied in its effects on the health of the host and the alteration of the gut microbiome. Although the key pathobionts in many diseases are not identified, there is emerging evidence that the favorable effects of berberine on health are mediated by modulating the gut microbiome, suggesting the existence of uncovered pathobionts in these diseases. Besides berberine, there are many other narrow-spectrum antibiotic agents naturally made by plants and bacteria, which may serve as tools to modulate the gut microbiome in the host. It would be interesting to investigate their effects on the health of the host and the role of fan-altered microbiome in these effects.

Among the five categories of antimicrobial agents reviewed in this article, STAMPs, phages, and targeted drug delivery systems can be designed to precisely target these pathobionts without disturbing normal flora in the gut. These strategies have the potential to achieve a higher level of precision on targeted bacteria than natural narrow-spectrum antimicrobial agents.

Besides the strategies reviewed in this article, probiotic and engineered bacteria have also been explored in modulating the gut microbiome. Effects of probiotics on the gut microbiome have been extensively reviewed (George Kerry et al., 2018; Fuenzalida et al., 2021) and engineered bacteria is a complicated topic. When designing such engineered bacteria, multiple factors need to be considered including the selection of chassis bacteria, control circuits, secretion strategies, and payload proteins (see review: Mays and Nair, 2018; Aggarwal et al., 2020; Brennan, 2021; Lynch et al., 2022; Shen et al., 2022).

For the pathobionts-targeting therapeutics, the detection of pathobionts is a challenge. One of the best detection methods of pathobionts is qPCR against their “virulence factors.” For example, the detection of Enterotoxigenic *Bacteroides fragilis* (ETBF) can be based on the qPCR of *B. fragilis* toxin (BFT) or fragilysin. As such, the understanding of the pathogenicity of pathobionts is crucial for developing detection methods. This is different from classical pathogens, in which a full understanding of the pathogenicity is often not required to develop detection methods against them.

To conclude, given the emerging discoveries on pathobionts and their pathogenesis, we expect as our knowledge of the human microbiome increases targeted antimicrobial agents will emerge as a new generation of drugs to treat microbiome-involved diseases.

## Author Contributions

All authors listed have made a substantial, direct, and intellectual contribution to the work and approved it for publication.

## Funding

This work is supported by the National Key Research and Development Program of China (2020YFA0907800), Shenzhen Science and Technology Innovation Program (KQTD20200820145822023), and the National Natural Science Foundation of China (Grant Nos. 31900056 and 32000096).

## Conflict of Interest

The authors declare that the research was conducted in the absence of any commercial or financial relationships that could be construed as a potential conflict of interest.

## Publisher’s Note

All claims expressed in this article are solely those of the authors and do not necessarily represent those of their affiliated organizations, or those of the publisher, the editors and the reviewers. Any product that may be evaluated in this article, or claim that may be made by its manufacturer, is not guaranteed or endorsed by the publisher.

## References

[ref1] Adrián Calderon J. S. Dapello G. Gamboa E. Rosas J. Chávez J. Retuerto F.. (2019). Assessment of antibacterial and antifungal properties and In vivo cytotoxicity of Peruvian Passiflora mollisima. J. Contemp. Dent. Pract. 20, 145–151. doi: 10.5005/jp-journals-10024-2489, PMID: 31058627

[ref2] Aggarwal N. Breedon A. Davis C. Hwang I. Chang M. J. C. O. I. B. (2020). Engineering probiotics for therapeutic applications: recent examples and translational outlook. Curr. Opin. Biotechnol. 65, 171–179. doi: 10.1016/j.copbio.2020.02.016 32304955

[ref3] Ahmadova A. Todorov S. D. Hadji-Sfaxi I. Choiset Y. Rabesona H. Messaoudi S.. (2013). Antimicrobial and antifungal activities of *Lactobacillus curvatus* strain isolated from homemade Azerbaijani cheese. Anaerobe 20, 42–49. doi: 10.1016/j.anaerobe.2013.01.003, PMID: 23357316

[ref4] Alm R. A. Lahiri S. D. (2020). Narrow-spectrum antibacterial agents-benefits and challenges. Antibiotics 9:418. doi: 10.3390/antibiotics9070418 PMC7400354 32708925

[ref5] Altarac D. Gutch M. Mueller J. Ronsheim M. Tommasi R. Perros M. (2021). Challenges and opportunities in the discovery, development, and commercialization of pathogen-targeted antibiotics. Drug Discov. Today 26, 2084–2089. doi: 10.1016/j.drudis.2021.02.014, PMID: 33610472

[ref6] Aresti Sanz J. El Aidy S. (2019). Microbiota and gut neuropeptides: a dual action of antimicrobial activity and neuroimmune response. Psychopharmacology 236, 1597–1609. doi: 10.1007/s00213-019-05224-0, PMID: 30997526 PMC6598950

[ref7] Avis T. Wilson F. X. Khan N. Mason C. S. Powell D. J. (2021). Targeted microbiome-sparing antibiotics. Drug Discov. Today 26, 2198–2203. doi: 10.1016/j.drudis.2021.07.016, PMID: 34329771

[ref8] Baindara P. Singh N. Ranjan M. Nallabelli N. Chaudhry V. Pathania G. L.. (2016). Laterosporulin10: a novel defensin like class IId bacteriocin from Brevibacillus sp. strain SKDU10 with inhibitory activity against microbial pathogens. Microbiology 162, 1286–1299. doi: 10.1099/mic.0.000316, PMID: 27267959

[ref9] Bhattacharjya S. Straus S. K. (2020). Design, engineering and discovery of novel α-helical and β-boomerang antimicrobial peptides against drug resistant Bacteria. Int. J. Mol. Sci. 21:5773. doi: 10.3390/ijms21165773, PMID: 32796755 PMC7460851

[ref10] Birri D. J. Brede D. A. Forberg T. Holo H. Nes I. F. (2010). Molecular and genetic characterization of a novel bacteriocin locus in *Enterococcus avium* isolates from infants. Appl. Environ. Microbiol. 76, 483–492. doi: 10.1128/AEM.01597-09, PMID: 19933345 PMC2805230

[ref11] Borrero J. Brede D. A. Skaugen M. Diep D. B. Herranz C. Nes I. F.. (2011). Characterization of garvicin ML, a novel circular bacteriocin produced by *Lactococcus garvieae* DCC43, isolated from mallard ducks (*Anas platyrhynchos*). Appl. Environ. Microbiol. 77, 369–373. doi: 10.1128/AEM.01173-10, PMID: 21057028 PMC3019728

[ref12] Bouttefroy A. Linder M. Milliere J. B. (2000). Predictive models of the combined effects of curvaticin 13, NaCl and pH on the behaviour of *Listeria monocytogenes* ATCC 15313 in broth. J. Appl. Microbiol. 88, 919–929. doi: 10.1046/j.1365-2672.2000.01053.x, PMID: 10849167

[ref13] Brennan A. J. S. B. (2021). Development of synthetic biotics as treatment for human diseases. Synth. Biol. 7:ysac001. doi: 10.1093/synbio/ysac001 PMC8944296 35350191

[ref14] Brennan C. A. Garrett W. S. (2019). *Fusobacterium nucleatum* - symbiont, opportunist and oncobacterium. Nat. Rev. Microbiol. 17, 156–166. doi: 10.1038/s41579-018-0129-6, PMID: 30546113 PMC6589823

[ref15] Cao Y. Wang Z. Yan Y. Ji L. He J. Xuan B.. (2021). Enterotoxigenic *Bacteroides fragilis* promotes intestinal inflammation and malignancy by inhibiting exosome-packaged miR-149-3p. Gastroenterology 161:e1512. doi: 10.1053/j.gastro.2021.08.003 34371001

[ref16] Carasso S. Hajjo H. Geva-Zatorsky N. (2020). Phage-Bacteria associations: analyze. Match. Develop Therapies. Cell Host. Microbe. 28, 353–355. doi: 10.1016/j.chom.2020.08.009, PMID: 32910916

[ref17] Chandra H. Sharma K. K. Tuovinen O. H. Sun X. Shukla P. (2021). Pathobionts: mechanisms of survival, expansion, and interaction with host with a focus on Clostridioides difficile. Gut Microbes 13:1979882. doi: 10.1080/19490976.2021.1979882, PMID: 34724858 PMC8565823

[ref18] Chen Y. S. Wu H. C. Kuo C. Y. Chen Y. W. Ho S. Yanagida F. (2018). Leucocin C-607, a novel Bacteriocin from the multiple-Bacteriocin-producing *Leuconostoc pseudomesenteroides* 607 isolated from persimmon. Probiotics. Antimicrob. Proteins 10, 148–156. doi: 10.1007/s12602-017-9359-6, PMID: 29177756

[ref19] Chen J. Zou X. (2019). Self-assemble peptide biomaterials and their biomedical applications. Bioact. Mater. 4, 120–131. doi: 10.1016/j.bioactmat.2019.01.002, PMID: 31667440 PMC6812166

[ref20] Cheng H. Liu J. Tan Y. Feng W. Peng C. (2021). Interactions between gut microbiota and berberine, a necessary procedure to understand the mechanisms of berberine. J. Pharmaceut. Analysis. doi: 10.1016/j.jpha.2021.10.003 PMC9463479 36105164

[ref21] Chepkirui C. Cheng T. Sum W. C. Matasyoh J. C. Decock C. Praditya D. F.. (2019). Skeletocutins A-L: antibacterial agents from the Kenyan Wood-inhabiting Basidiomycete, Skeletocutis sp. J. Agric. Food Chem. 67, 8468–8475. doi: 10.1021/acs.jafc.9b02598, PMID: 31310114

[ref22] Chhibber S. Kaur T. Sandeep K. (2013). Co-therapy using lytic bacteriophage and linezolid: effective treatment in eliminating methicillin resistant *Staphylococcus aureus* (MRSA) from diabetic foot infections. PLoS One 8:e56022. doi: 10.1371/journal.pone.0056022, PMID: 23418497 PMC3572146

[ref23] Chou S. Li Q. Wu H. Li J. Chang Y. F. Shang L.. (2021). Selective antifungal activity and fungal biofilm inhibition of tryptophan center symmetrical short peptide. Int. J. Mol. Sci. 22:8231. doi: 10.3390/ijms22158231, PMID: 34360998 PMC8348200

[ref24] Chou S. Shao C. Wang J. Shan A. Xu L. Dong N.. (2016). Short, multiple-stranded beta-hairpin peptides have antimicrobial potency with high selectivity and salt resistance. Acta Biomater. 30, 78–93. doi: 10.1016/j.actbio.2015.11.002, PMID: 26546414

[ref25] Chou S. Wang J. Shang L. Akhtar M. U. Wang Z. Shi B.. (2019). Short, symmetric-helical peptides have narrow-spectrum activity with low resistance potential and high selectivity. Biomater. Sci. 7, 2394–2409. doi: 10.1039/C9BM00044E, PMID: 30919848

[ref26] Chow J. Tang H. Mazmanian S. K. (2011). Pathobionts of the gastrointestinal microbiota and inflammatory disease. Curr. Opin. Immunol. 23, 473–480. doi: 10.1016/j.coi.2011.07.010, PMID: 21856139 PMC3426444

[ref27] Chung L. Thiele Orberg E. Geis A. L. Chan J. L. Fu K. Destefano Shields C. E.. (2018). *Bacteroides fragilis* toxin coordinates a pro-carcinogenic inflammatory Cascade via targeting of colonic epithelial cells. Cell Host Microbe 23:e205. doi: 10.1016/j.chom.2018.02.004 PMC6469393 29544099

[ref28] Colakoglu M. Xue J. Trajkovski M. (2020). Bacteriophage prevents alcoholic liver disease. Cell 180, 218–220. doi: 10.1016/j.cell.2019.12.034, PMID: 31978341

[ref29] de Leeuw E. Burks S. R. Li X. Kao J. P. Lu W. (2007). Structure-dependent functional properties of human Defensin 5. FEBS Lett. 581, 5733–5520. doi: 10.1016/j.febslet.2006.12.036, PMID: 17250830 PMC1832120

[ref30] Dijksteel G. S. Ulrich M. M. W. Middelkoop E. Boekema B. (2021). Review: lessons learned From clinical trials using antimicrobial peptides (AMPs). Front. Microbiol. 12:616979. doi: 10.3389/fmicb.2021.616979, PMID: 33692766 PMC7937881

[ref31] Duan Y. Llorente C. Lang S. Brandl K. Chu H. Jiang L.. (2019). Bacteriophage targeting of gut bacterium attenuates alcoholic liver disease. Nature 575, 505–511. doi: 10.1038/s41586-019-1742-x, PMID: 31723265 PMC6872939

[ref32] Eckert R. Brady K. M. Greenberg E. P. Qi F. Yarbrough D. K. He J.. (2006). Enhancement of antimicrobial activity against *pseudomonas aeruginosa* by coadministration of G10KHc and tobramycin. Antimicrob. Agents Chemother. 50, 3833–3838. doi: 10.1128/AAC.00509-06, PMID: 16940063 PMC1635211

[ref33] Eskandari S. Guerin T. Toth I. Stephenson R. J. (2017). Recent advances in self-assembled peptides: implications for targeted drug delivery and vaccine engineering. Adv. Drug Deliv. Rev. 110–111, 169–187. doi: 10.1016/j.addr.2016.06.013, PMID: 27356149

[ref34] Fang Y. Zhang J. Zhu S. He M. Ma S. Jia Q.. (2021a). Berberine ameliorates ovariectomy-induced anxiety-like behaviors by enrichment in equol generating gut microbiota. Pharmacol. Res. 165:105439. doi: 10.1016/j.phrs.2021.105439, PMID: 33493658

[ref35] Fang Y. Zhu Y. Li L. Lai Z. Dong N. Shan A. (2021b). Biomaterial-interrelated bacterial sweeper: simplified self-assembled Octapeptides with double-layered Trp zipper induces membrane destabilization and bacterial apoptosis-Like death. Small Methods 5:e2101304. doi: 10.1002/smtd.202101304 34928043

[ref36] Ferreira M. C. Cantrell C. L. Wedge D. E. Goncalves V. N. Jacob M. R. Khan S.. (2017). Antimycobacterial and antimalarial activities of endophytic fungi associated with the ancient and narrowly endemic neotropical plant Vellozia gigantea from Brazil. Mem. Inst. Oswaldo Cruz 112, 692–697. doi: 10.1590/0074-02760170144, PMID: 28953997 PMC5607518

[ref37] Fong F. L. Shah N. P. Kirjavainen P. El-Nezami H. (2016). Mechanism of action of probiotic Bacteria on intestinal and systemic immunities and antigen-presenting cells. Int. Rev. Immunol. 35, 179–188. doi: 10.3109/08830185.2015.1096937, PMID: 26606641

[ref38] Fuenzalida C. Dufeu M. Poniachik J. Roblero J. Valenzuela-Pérez L. Beltrán C. J. F. I. P. (2021). Probiotics-based treatment as an integral approach for alcohol use disorder in alcoholic liver disease. Front. Pharmacol. 12:729950. doi: 10.3389/fphar.2021.729950 34630107 PMC8497569

[ref39] George Kerry R. Patra J. K. Gouda S. Park Y. Shin H. S. Das G. (2018). Benefaction of probiotics for human health: a review. J. Food Drug Anal. 26, 927–939. doi: 10.1016/j.jfda.2018.01.002, PMID: 29976412 PMC9303019

[ref40] Gill T. Rosenbaum J. J. F. I. I. (2020). Putative Pathobionts in HLA-B27-Associated Spondyloarthropathy. Front. Immunol. 11:586494. doi: 10.3389/fimmu.2020.586494 33537028 PMC7848169

[ref41] Gonzalez-Lamothe R. Mitchell G. Gattuso M. Diarra M. S. Malouin F. Bouarab K. (2009). Plant antimicrobial agents and their effects on plant and human pathogens. Int. J. Mol. Sci. 10, 3400–3419. doi: 10.3390/ijms10083400, PMID: 20111686 PMC2812829

[ref42] Gordillo F. L. Altamirano A. Barra J. J. (2019). Phage therapy in the Postantibiotic era. Clinical Microbiol. Rev. 32:18. doi: 10.1128/CMR.00066-18, PMID: 30651225 PMC6431132

[ref43] Gowd V. Karim N. Shishir M. R. I. Xie L. Chen W. (2019). Dietary polyphenols to combat the metabolic diseases via altering gut microbiota. Trends Food Sci. Technol. 93, 81–93. doi: 10.1016/j.tifs.2019.09.005

[ref44] Gu Liu C. Green S. I. Min L. Clark J. R. Salazar K. C. Terwilliger A. L.. (2020). Phage-antibiotic synergy is driven by a unique combination of antibacterial mechanism of action and stoichiometry. MBio 11:e01462-20 doi: 10.1128/mBio.01462-20 32753497 PMC7407087

[ref45] Guo L. Mclean J. S. Yang Y. Eckert R. Kaplan C. W. Kyme P.. (2015). Precision-guided antimicrobial peptide as a targeted modulator of human microbial ecology. Proc. Natl. Acad. Sci. U. S. A. 112, 7569–7574. doi: 10.1073/pnas.1506207112, PMID: 26034276 PMC4475959

[ref46] Hart P. Wood T. M. Tehrani K. Van Harten R. M. Sleszynska M. Rentero Rebollo I.. (2017). De novo identification of lipid II binding lipopeptides with antibacterial activity against vancomycin-resistant bacteria. Chem. Sci. 8, 7991–7997. doi: 10.1039/C7SC03413J, PMID: 29568446 PMC5853558

[ref47] Hashemi M. M. Rovig J. Bateman J. Holden B. S. Modelzelewski T. Gueorguieva I.. (2018). Preclinical testing of a broad-spectrum antimicrobial endotracheal tube coated with an innate immune synthetic mimic. J. Antimicrob. Chemother. 73, 143–150. doi: 10.1093/jac/dkx347, PMID: 29029265 PMC5890737

[ref48] Hsu C. L. Duan Y. Fouts D. E. Schnabl B. (2021). Intestinal virome and therapeutic potential of bacteriophages in liver disease. J. Hepatol. 75, 1465–1475. doi: 10.1016/j.jhep.2021.08.003, PMID: 34437908 PMC8929164

[ref49] Huang P. Xie F. Ren B. Wang Q. Wang J. Wang Q.. (2016). Anti-MRSA and anti-TB metabolites from marine-derived Verrucosispora sp. MS100047. Appl. Microbiol. Biotechnol. 100, 7437–7447. doi: 10.1007/s00253-016-7406-y, PMID: 26975378

[ref50] Huo L. Huang X. Ling J. Liu H. Liu J. (2018). Selective activities of STAMPs against *Streptococcus mutans*. Exp. Ther. Med. 15, 1886–1893. doi: 10.3892/etm.2017.5631, PMID: 29434779 PMC5776616

[ref51] Hussein H. A. Abdullah M. A. (2021). Novel drug delivery systems based on silver nanoparticles, hyaluronic acid, lipid nanoparticles and liposomes for cancer treatment. Appl. Nano, 1–26. doi: 10.1007/s13204-021-02018-9

[ref52] Iftekhar A. Berger H. Bouznad N. Heuberger J. Boccellato F. Dobrindt U.. (2021). Genomic aberrations after short-term exposure to colibactin-producing *E. coli* transform primary colon epithelial cells. Nat. Commun. 12:1003. doi: 10.1038/s41467-021-21162-y, PMID: 33579932 PMC7881031

[ref53] Jennings A. Koch M. Bang C. Franke A. Lieb W. Cassidy A. (2021). Microbial diversity and abundance of Parabacteroides mediate the associations Between higher intake of flavonoid-rich foods and lower blood pressure. Hypertension 78, 1016–1026. doi: 10.1161/HYPERTENSIONAHA.121.17441, PMID: 34420369

[ref54] Jeong Y. J. Moon G. S. (2015). Antilisterial Bacteriocin from *Lactobacillus rhamnosus* CJNU 0519 presenting a narrow antimicrobial Spectrum. Korean J. Food Sci. Anim. Resour. 35, 137–142. doi: 10.5851/kosfa.2015.35.1.137, PMID: 26761811 PMC4682500

[ref55] Kozarski M. Klaus A. Vunduk J. Zizak Z. Niksic M. Jakovljevic D.. (2015). Nutraceutical properties of the methanolic extract of edible mushroom Cantharellus cibarius (fries): primary mechanisms. Food Funct. 6, 1875–1886. doi: 10.1039/C5FO00312A, PMID: 25943486

[ref56] Kumariya R. Garsa A. K. Rajput Y. S. Sood S. K. Akhtar N. Patel S. (2019). Bacteriocins: classification, synthesis, mechanism of action and resistance development in food spoilage causing bacteria. Microb. Pathog. 128, 171–177. doi: 10.1016/j.micpath.2019.01.002, PMID: 30610901

[ref57] Le H. Arnoult C. De E. Schapman D. Galas L. Le Cerf D.. (2021). Antibody-conjugated Nanocarriers for targeted antibiotic delivery: application in the treatment of bacterial biofilms. Biomacromolecules 22, 1639–1653. doi: 10.1021/acs.biomac.1c00082, PMID: 33709706

[ref58] Lei M. Jayaraman A. Van Deventer J. A. Lee K. (2021). Engineering selectively targeting antimicrobial peptides. Annu. Rev. Biomed. Eng. 23, 339–357. doi: 10.1146/annurev-bioeng-010220-095711, PMID: 33852346 PMC9017812

[ref59] Li J. L. Jiang X. Liu X. He C. Di Y. Lu S.. (2019). Antibacterial anthraquinone dimers from marine derived fungus Aspergillus sp. Fitoterapia 133, 1–4. doi: 10.1016/j.fitote.2018.11.015, PMID: 30543983

[ref60] Li J. Shang L. Lan J. Chou S. Feng X. Shi B.. (2020). Targeted and intracellular antibacterial activity against *S. agalactiae* of the chimeric peptides based on pheromone and cell-penetrating peptides. ACS Appl. Mater. Interfaces 12, 44459–44474. doi: 10.1021/acsami.0c12226, PMID: 32924418

[ref61] Li X. Su C. Jiang Z. Yang Y. Zhang Y. Yang M.. (2021). Berberine attenuates choline-induced atherosclerosis by inhibiting trimethylamine and trimethylamine-N-oxide production via manipulating the gut microbiome. NPJ Biofilms Microbiomes 7:36. doi: 10.1038/s41522-021-00205-8, PMID: 33863898 PMC8052457

[ref62] Lin Yuan S. S.-Y. Jian-Dong J. (2018). Antibacterial activity of berberine. Acta Pharm. Sin. 12, 163–168.

[ref63] Lynch J. Goers L. Lesser C. J. T. I. P. S. (2022). Emerging strategies for engineering *Escherichia coli* Nissle 1917-based therapeutics. Trends Pharmacol. Sci. doi: 10.1016/j.tips.2022.02.002, PMID: 35232591 PMC9378478

[ref64] Maraming P. Klaynongsruang S. Boonsiri P. Peng S. F. Daduang S. Leelayuwat C.. (2019). The cationic cell-penetrating KT2 peptide promotes cell membrane defects and apoptosis with autophagy inhibition in human HCT 116 colon cancer cells. J. Cell. Physiol. 234, 22116–22129. doi: 10.1002/jcp.28774, PMID: 31073999

[ref65] Martinet L. Naômé A. Deflandre B. Maciejewska M. Tellatin D. Tenconi E. (2019). A Single Biosynthetic Gene Cluster Is Responsible for the Production of Bagremycin Antibiotics and Ferroverdin Iron Chelators. Mbio 10:e01230-19. doi: 10.1128/mBio.01230-19 31409675 PMC6692506

[ref66] Martin-Visscher L. A. Yoganathan S. Sit C. S. Lohans C. T. Vederas J. C. (2011). The activity of bacteriocins from *Carnobacterium maltaromaticum* UAL307 against gram-negative bacteria in combination with EDTA treatment. FEMS Microbiol. Lett. 317, 152–159. doi: 10.1111/j.1574-6968.2011.02223.x, PMID: 21255070

[ref67] Masuda Y. Ono H. Kitagawa H. Ito H. Mu F. Sawa N.. (2011). Identification and characterization of leucocyclicin Q, a novel cyclic bacteriocin produced by *Leuconostoc mesenteroides* TK41401. Appl. Environ. Microbiol. 77, 8164–8170. doi: 10.1128/AEM.06348-11, PMID: 21948835 PMC3209004

[ref68] Maxson T. Mitchell D. A. (2016). Targeted treatment for bacterial infections: prospects for pathogen-specific antibiotics coupled with rapid diagnostics. Tetrahedron 72, 3609–3624. doi: 10.1016/j.tet.2015.09.069, PMID: 27429480 PMC4941824

[ref69] Mays Z. J. Nair N. U. (2018). Synthetic biology in probiotic lactic acid bacteria: at the frontier of living therapeutics. Curr. Opin. Biotechnol. 53, 224–231. doi: 10.1016/j.copbio.2018.01.028, PMID: 29550614 PMC6139064

[ref70] Mccarville J. L. Caminero A. Verdu E. F. (2016). Novel perspectives on therapeutic modulation of the gut microbiota. Ther. Adv. Gastroenterol. 9, 580–593. doi: 10.1177/1756283X16637819, PMID: 27366225 PMC4913331

[ref71] Meade E. Slattery M. A. Garvey M. (2020). Bacteriocins, potent antimicrobial peptides and the fight against multi drug resistant species: resistance is futile? Antibiotics 9:10032. doi: 10.3390/antibiotics9010032 PMC7168330 31963311

[ref72] Mengxin Geng A. Smitha L. (2018). Modifying the lantibiotic mutacin 1140 for increased yield, activity, and stability. Appl. Env. Microbiol. 84:18. doi: 10.1128/AEM.00830-18 PMC6052277 29776930

[ref73] Mishra B. Wang G. (2012). Ab initio design of potent anti-MRSA peptides based on database filtering technology. J. Am. Chem. Soc. 134, 12426–12429. doi: 10.1021/ja305644e, PMID: 22803960 PMC3412535

[ref74] Moll G. N. Konings W. N. Driessen A. J. (1999). Bacteriocins: mechanism of membrane insertion and pore formation. Antonie Van Leeuwenhoek 76, 185–198. doi: 10.1023/A:1002002718501, PMID: 10532378

[ref75] Morrisette T. Kebriaei R. Lev K. L. Morales S. Rybak M. J. (2020). Bacteriophage therapeutics: a primer for clinicians on phage-antibiotic combinations. Pharmacotherapy 40, 153–168. doi: 10.1002/phar.2358, PMID: 31872889

[ref76] Nale J. Y. Redgwell T. A. Millard A. Clokie M. R. J. (2018). Efficacy of an optimised bacteriophage cocktail to clear *Clostridium difficile* in a batch fermentation model. Antibiotics 7:10013. doi: 10.3390/antibiotics7010013 PMC5872124 29438355

[ref77] Osmanagaoglu O. Kiran F. (2011). Evidence for a chromosomally determined mesenterocin, a bacteriocin produced by *Leuconostoc mesenteroides* subsp. mesenteroides OZ. J. Basic Microbiol. 51, 279–288. doi: 10.1002/jobm.201000240, PMID: 21298683

[ref78] Ostaff M. J. Stange E. F. Wehkamp J. (2013). Antimicrobial peptides and gut microbiota in homeostasis and pathology. EMBO Mol. Med. 5, 1465–1483. doi: 10.1002/emmm.201201773, PMID: 24039130 PMC3799574

[ref79] Padhi S. Masi M. Panda S. K. Luyten W. Cimmino A. Tayung K.. (2020). Antimicrobial secondary metabolites of an endolichenic Aspergillus Niger isolated from lichen thallus of Parmotrema ravum. Nat. Prod. Res. 34, 2573–2580. doi: 10.1080/14786419.2018.1544982, PMID: 30600725

[ref80] Phulen Sarma S. M. Prakash A. Medhi B. (2018). Specifically targeted antimicrobial peptides: a new and promising avenue in selective antimicrobial therapy. Indian J. Pharmacol. 50, 1–3. 29861521 10.4103/ijp.IJP_218_18 PMC5954627

[ref81] Pleguezuelos-Manzano C. Puschhof J. Rosendahl Huber A. Van Hoeck A. Wood H. M. Nomburg J.. (2020). Mutational signature in colorectal cancer caused by genotoxic pks(+) *E. coli*. Nature 580, 269–273. doi: 10.1038/s41586-020-2080-8 32106218 PMC8142898

[ref82] Qin Y. Wang Y. He Y. Zhang Y. She Q. Chai Y.. (2019). Characterization of Subtilin L-Q11, a novel class I Bacteriocin synthesized by *Bacillus subtilis* L-Q11 isolated From orchard soil. Front. Microbiol. 10:484. doi: 10.3389/fmicb.2019.00484, PMID: 30930878 PMC6429107

[ref83] Qiu X. Q. Wang H. Lu X. F. Zhang J. Li S. F. Cheng G.. (2003). An engineered multidomain bactericidal peptide as a model for targeted antibiotics against specific bacteria. Nat. Biotechnol. 21, 1480–1485. doi: 10.1038/nbt913, PMID: 14625561

[ref84] Ren S. Cai Y. Hu S. Liu J. Zhao Y. Ding M.. (2021). Berberine exerts anti-tumor activity in diffuse large B-cell lymphoma by modulating c-myc/CD47 axis. Biochem. Pharmacol. 188:114576. doi: 10.1016/j.bcp.2021.114576, PMID: 33930347

[ref85] Riglar D. T. Silver P. A. (2018). Engineering bacteria for diagnostic and therapeutic applications. Nat. Rev. Microbiol. 16, 214–225. doi: 10.1038/nrmicro.2017.172 29398705

[ref86] Ríos Colombo N. S. Chalón M. C. Dupuy F. G. Gonzalez C. F. Bellomio A. (2019). The case for class II bacteriocins: A biophysical approach using "suicide probes" in receptor-free hosts to study their mechanism of action. Biochimie 165, 183–195. doi: 10.1016/j.biochi.2019.07.024, PMID: 31381962

[ref87] Romani-Perez M. Agusti A. Sanz Y. (2017). Innovation in microbiome-based strategies for promoting metabolic health. Curr. Opin. Clin. Nutr. Metab. Care 20, 484–491. doi: 10.1097/MCO.0000000000000419, PMID: 28862999

[ref88] Roncevic T. Vukicevic D. Krce L. Benincasa M. Aviani I. Maravic A.. (2019). Selection and redesign for high selectivity of membrane-active antimicrobial peptides from a dedicated sequence/function database. Biochim. Biophys. Acta Biomembr. 1861, 827–834. doi: 10.1016/j.bbamem.2019.01.017, PMID: 30710514

[ref89] Rubinstein M. R. Wang X. Liu W. Hao Y. Cai G. Han Y. W. (2013). *Fusobacterium nucleatum* promotes colorectal carcinogenesis by modulating E-cadherin/beta-catenin signaling via its FadA adhesin. Cell Host Microbe 14, 195–206. doi: 10.1016/j.chom.2013.07.012, PMID: 23954158 PMC3770529

[ref90] Rufino A. T. Costa V. M. Carvalho F. Fernandes E. (2021). Flavonoids as antiobesity agents: A review. Med. Res. Rev. 41, 556–585. doi: 10.1002/med.21740, PMID: 33084093

[ref91] Shen H. Zhao Z. Zhao Z. Chen Y. Zhang L. J. I. J. O. M. S. (2022). Native and engineered probiotics: promising agents against related systemic and intestinal diseases. Int. J. Mol. Sci. 23:594, 23. doi: 10.3390/ijms23020594 35054790 PMC8775704

[ref92] Sivieri K. Bassan J. Peixoto G. Monti R. (2017). Gut microbiota and antimicrobial peptides. Curr. Opin. Food Sci. 13, 56–62. doi: 10.1016/j.cofs.2017.02.010

[ref93] Slade D. J. (2021). New roles for *Fusobacterium nucleatum* in Cancer: target the Bacteria, host, or Both? Trends Cancer 7, 185–187. doi: 10.1016/j.trecan.2020.11.006, PMID: 33309240

[ref94] Soltani S. Hammami R. Cotter P. D. Rebuffat S. Said L. B. Gaudreau H.. (2021). Bacteriocins as a new generation of antimicrobials: toxicity aspects and regulations. FEMS Microbiol. Rev. 45:39. doi: 10.1093/femsre/fuaa039, PMID: 32876664 PMC7794045

[ref95] Sullivan R. Santarpia P. Lavender S. Gittins E. Liu Z. Anderson M. H.. (2011). Clinical efficacy of a specifically targeted antimicrobial peptide mouth rinse: targeted elimination of Streptococcus mutans and prevention of demineralization. Caries Res. 45, 415–428. doi: 10.1159/000330510, PMID: 21860239 PMC3169368

[ref96] Tan P. Fu H. Ma X. (2021). Design, optimization, and nanotechnology of antimicrobial peptides: From exploration to applications. Nano Today 39:101229. doi: 10.1016/j.nantod.2021.101229

[ref97] Ugurlu T. Turkoglu M. Gurer U. S. Akarsu B. G. (2007). Colonic delivery of compression coated nisin tablets using pectin/HPMC polymer mixture. Eur. J. Pharm. Biopharm. 67, 202–210. doi: 10.1016/j.ejpb.2007.01.016, PMID: 17337171

[ref98] Vaičikauskaitė M. Ger M. Valius M. Maneikis A. Lastauskienė E. Kalėdienė L.. (2019). Geobacillin 26 - high molecular weight bacteriocin from a thermophilic bacterium. Int. J. Biol. Macromol. 141, 333–344. doi: 10.1016/j.ijbiomac.2019.09.047 31499103

[ref99] Villarroel J. Larsen M. V. Kilstrup M. Nielsen M. (2017). Metagenomic analysis of therapeutic PYO phage cocktails from 1997 to 2014. Viruses 9:328. doi: 10.3390/v9110328, PMID: 29099783 PMC5707535

[ref100] Wahida A. Tang F. Barr J. J. (2021). Rethinking phage-bacteria-eukaryotic relationships and their influence on human health. Cell Host Microbe 29, 681–688. doi: 10.1016/j.chom.2021.02.007, PMID: 33735620

[ref101] Wang J. Chou S. Xu L. Zhu X. Dong N. Shan A.. (2015). High specific selectivity and membrane-active mechanism of the synthetic centrosymmetric alpha-helical peptides with Gly-Gly pairs. Sci. Rep. 5:15963. doi: 10.1038/srep15963, PMID: 26530005 PMC4632126

[ref102] Wang G. Li X. Wang Z. (2009). APD2: the updated antimicrobial peptide database and its application in peptide design. Nucleic Acids Res. 37, D933–D937. doi: 10.1093/nar/gkn823, PMID: 18957441 PMC2686604

[ref103] Xiong M. Bao Y. Xu X. Wang H. Han Z. Wang Z.. (2017). Selective killing of *Helicobacter pylori* with pH-responsive helix-coil conformation transitionable antimicrobial polypeptides. Proc. Natl. Acad. Sci. U. S. A. 114, 12675–12680. doi: 10.1073/pnas.1710408114, PMID: 29133389 PMC5715757

[ref104] Xu L. Shao C. Li G. Shan A. Chou S. Wang J.. (2020). Conversion of broad-Spectrum antimicrobial peptides into species-specific antimicrobials capable of precisely targeting pathogenic Bacteria. Sci. Rep. 10:944. doi: 10.1038/s41598-020-58014-6, PMID: 31969663 PMC6976587

[ref105] Xue Dong P. P. Zheng D.-W. Bao P. Zeng X. Zhang X.-Z. (2020). Bioinorganic hybrid bacteriophage for modulation of intestinal microbiota to remodel tumor-immune microenvironment against colorectal cancer. Sci. Adv. 6:1590. doi: 10.1126/sciadv.aba1590 PMC7228756 32440552

[ref106] Yachida S. Mizutani S. Shiroma H. Shiba S. Nakajima T. Sakamoto T.. (2019). Metagenomic and metabolomic analyses reveal distinct stage-specific phenotypes of the gut microbiota in colorectal cancer. Nat. Med. 25, 968–976. doi: 10.1038/s41591-019-0458-7, PMID: 31171880

[ref107] Yang S. Han X. Yang Y. Qiao H. Yu Z. Liu Y.. (2018). Bacteria-targeting nanoparticles with microenvironment-responsive antibiotic release to eliminate intracellular Staphylococcus aureus and associated infection. ACS Appl. Mater. Interfaces 10, 14299–14311. doi: 10.1021/acsami.7b15678, PMID: 29633833

[ref108] Yount N. Y. Weaver D. C. Lee E. Y. Lee M. W. Wang H. Chan L. C.. (2019). Unifying structural signature of eukaryotic alpha-helical host defense peptides. Proc. Natl. Acad. Sci. U. S. A. 116, 6944–6953. doi: 10.1073/pnas.1819250116, PMID: 30877253 PMC6452647

[ref109] Zhang Y. Gu Y. Ren H. Wang S. Zhong H. Zhao X.. (2020b). Gut microbiome-related effects of berberine and probiotics on type 2 diabetes (the PREMOTE study). Nat. Commun. 11:5015. doi: 10.1038/s41467-020-18414-8, PMID: 33024120 PMC7538905

[ref110] Zhang Y. Li C. X. Zhang X. Z. (2021). Bacteriophage-mediated modulation of microbiota for diseases treatment. Adv. Drug Deliv. Rev. 176:113856. doi: 10.1016/j.addr.2021.113856, PMID: 34237403

[ref111] Zhang L. Wu X. Yang R. Chen F. Liao Y. Zhu Z.. (2020a). Effects of Berberine on the gastrointestinal microbiota. Front. Cell. Infect. Microbiol. 10:588517. doi: 10.3389/fcimb.2020.588517 33680978 PMC7933196

[ref112] Zhao Y. Jiang Q. (2021). Roles of the polyphenol-gut microbiota interaction in alleviating colitis and preventing colitis-associated colorectal Cancer. Adv. Nutr. 12, 546–565. doi: 10.1093/advances/nmaa104, PMID: 32905583 PMC8009754

